# Supplementary material for: Genome-wide promoter responses to CRISPR perturbations of regulators reveal regulatory networks in Escherichia coli
Source: Nat Commun. 2023 Sep 16;14:5757. doi: 10.1038/s41467-023-41572-4 (PMC10505187; doi:10.1038/s41467-023-41572-4)
Supplement: Supplementary file 1 — Supplementary Information [file 41467_2023_41572_MOESM1_ESM.pdf]

Supplementary Information for

Genome-wide promoter responses to CRISPR perturbations of regulators reveal regulatory networks in *Escherichia coli*

Authors: Yichao Han[1], Wanji Li[1], Alden Filko[1], Jingyao Li[1], Fuzhong Zhang[1-3,] *

Affiliations:

[1]Department of Energy, Environmental and Chemical Engineering,

[2] Division of Biological & Biomedical Sciences,

[3] Institute of Materials Science & Engineering,

Washington University in St. Louis, Saint Louis, Missouri, USA.

*e-mail: fzhang@seas.wustl.edu

Table of Contents

Supplementary Notes: **4**

Supplementary Note 1. Evaluating the efficiency of CRISPRi repression 4

Supplementary Note 2. Global effects of TFs and definition of variable promoters 4

Supplementary Note 3. Mutual regulation and repressilator from different growth conditions 5

Supplementary Note 4. Context-dependent regulation of *arnB and fadE* promoters 5

Supplementary Note 5. Estimation of false positive and false negative rates 6

Supplementary Note 6. Choice of replication origin 7

Supplementary Figures: **8**

Supplementary Figure 1. FACS gate settings and sequencing results of PPTP-seq. 9

Supplementary Figure 2. Representative PPTP-seq results. 10

Supplementary Figure 3. Data quality of PPTP-seq performed at different conditions. 11

Supplementary Figure 4. Promoter activity fold changes by TFKD. 12

Supplementary Figure 5. Evaluation of CRISPRi repression efficiency. 14

Supplementary Figure 6. Benchmarks of PPTP-seq on five random promoters. 15

Supplementary Figure 7. Correlation of PPTP-seq data with published data sets. 17

Supplementary Figure 8. Validation of TF auto-regulatory responses 18

Supplementary Figure 9. Promoter activity changes in one-carbon metabolism. 19

Supplementary Figure 10. Context-dependent regulatory functions for promoters controlled by multiple TFs. ... 21

Supplementary Figure 11. Various types of regulatory architectures on promoters according to RegulonDB. 23

Supplementary Figure 12. Promoter responses by TFKD in LB and M9 glycerol media. 24

Supplementary Figure 13. Potential extensions of PPTP-seq 26

Supplementary Figure 14. Data quality filters improved data consistency 27

Supplementary Figure 15. Effects of plasmid copy number on cell growth 28

Supplementary Tables: **29**

Supplementary Table 1. Regulatory responses identified from PPTP-seq and known from RegulonDB 29

Supplementary Table 2. The number of responses for selected TFs in different conditions 30

Supplementary References 31

Supplementary Notes:

Supplementary Note 1. Evaluating the efficiency of CRISPRi repression

Two approaches were used to evaluate the repression efficiency of CRISPRi in our library. First, we examined sequences in the promoter library that can be directly targeted by sgRNAs, thus the CRISPRi repression efficiency of a TF can be estimated by the fold of repression on its own promoter. Totally 41 TFs have their promoters in our library, and 39 of them (95%) showed repression (Student's t-test P value < 0.05) (Supplementary Fig 5a). Second, additional RT-qPCR experiments were performed to examine the CRISPRi repression efficiency. The nine TFs that were used to examine individual responses (Supplementary Fig 6) and another five TFs (HipB, NarP, OmpR, YefM, and YeoB) randomly selected were chosen as representatives. The RT-qPCR results showed clear repression for 12 out of 14 TFs, representing 86% is effective (Supplementary Fig 5b).

Supplementary Note 2. Global effects of TFs and definition of variable promoters

A few TFs might cause global effects on the GFP expression level rather than TF-specific effects on promoter activities. Considering this factor, the global effect of a TF was quantified as the median fold change of all promoter activities caused by the TF, and this global effect was subtracted from the original fold change value to get the specific effect on a promoter. Promoters with at least one substantial specific effect (i.e., >1.7-fold change) from a TF on the promoter activity were defined as variable promoters. Among 978 promoters with significant activity change under at least one TFKD condition, we found 42 promoters with promoter activity changes by only global effects from TFs and classified them as constant promoters.

Supplementary Note 3. Mutual regulation and repressilator from different growth conditions

PPTP-seq date collected in LB medium revealed mutual regulation for two pairs of TFs: 1) UlaR represses *agaR*, and AgaR activates *ulaR*; 2) GlcC represses *allR*, and AllR represses *glcC.* AgaR activation of *ulaR*, and GlcC repression of *allR* are present in both M9 glucose and LB media, while UlaR repression of *agaR*, and AllR repression of *glcC* are absent in M9 glucose. We also found AllR, GlcC, and AtoC form a repressilator in LB: AllR represses *glcC*, GlcC represses *atoC*, and AtoC represses *allR*. But these regulations are absent in M9 glucose and M9 glycerol media. No binding evidence has been found for these regulatory responses, suggesting indirect interactions mediated by other molecules. We noticed that AllR and GlcC are regulated by glyoxylate and glycolate respectively[1,2]. Thus, genes in this repressilator may not have direct regulation and may display different dynamic properties with those from synthetic repressilators[3].

Supplementary Note 4. Context-dependent regulation of *arnB and fadE* promoters

arnB promoter controls the *arnBCADTEF* operon that mediates the biosynthesis and transfer of 4-amino-4-deoxy-L-arabinose to lipid A, which causes cell resistance to polymyxin. DAP-seq identified two binding sites on the *arnB* promoter, one for activator (BasR) and one for repressor (H-NS) with similar binding strength. However, PPTP-seq revealed that only knockdown of *hns*, but not *basR*, changed *arnB* promoter activity (Supplementary Fig. 10a). Our PPTP-seq results are consistent with those from TF titration, where only overexpression of H-NS, but not BasR, decreased *arnB* promoter activity (Supplementary Fig . 10b). We further ruled out the possibility of low BasR activity. Because BasR's activity can be enhanced by either Fe^{2+} or Fe^{3+} [4], we titrated both H-NS and BasR in the presence of these metal ions (Supplementary Fig. 10c, d). Under both conditions, *arnB* promoter's activity was not significantly changed. The results further confirmed

the context-dependent regulation where BasR does not regulate the *arnB* promoter due to repression of the *arnB* promoter by H-NS binding.

To investigate whether deactivating an active repressor on a promoter can make the promoter respond to other TFs, we selected the *fadE* promoter, which can be bound by two metabolite-responsive TFs, FadR and PdhR, according to DAP-seq results (Supplementary Fig. 10e). However, PPTP-seq revealed that *fadE* was up-regulated by the knockdown of only *fadR*, but not *pdhR*, in minimal glucose medium. Our PPTP-seq result is consistent with that from the tunable TF library, where overexpression of PdhR did not repress the *fadE* promoter (Supplementary Fig. 10f). Because PdhR binding to DNA can be potentially antagonized by high concentrations of pyruvate inside the cell[5]. We then tested whether the lack of activation by *pdhR* knockdown was caused by the high concentration of pyruvate. To do so, succinate was fed to cells to block pyruvate supply[6]. However, *fadE* promoter activity remained constant over a wide range of PdhR concentrations in succinate media (Supplementary Fig. 10g). Next, because the binding sites of FadR and PdhR on *fadE* promoter overlap, we tested whether FadR binding prevents PdhR from binding to the *fadE* promoter. To test so, FA was added to the growth medium to antagonize FadR binding to DNA[7–10]. Indeed, in both FA and glycerol/FA media, PdhR overexpression repressed *fadE* promoter by 6.4- and 5.0-fold, respectively (Supplementary Fig. 10h, i). Therefore, PdhR does not regulate *fadE* promoter in minimal glucose medium because FadR binding to *fadE* promoter interferes with PdhR binding.

Supplementary Note 5. Estimation of false positive and false negative rates

PPTP-seq is based on gene response to TF perturbation, thus false positives can be caused by off-target and polarity of CRISPRi (i.e., response to unexpected perturbations[11–14]). Previous genome-scale CRISPRi screens in *E. coli* showed an off-target probability of 10.7%[11] and a polarity

probability is 8.0%[12]. Our sgRNA design eliminated bad-seed effects [11] that cause off-target in the previous CRISPRi screens, thus is expected to have reduced off-target rate (<10.7%). Additionally, if the unexpectedly perturbed gene was not a TF gene, it is less likely to cause any promoter activity changes. Considering these, the expected false positive rate should be substantially less than 1-(1-10.7%)*(1-8%)=17.8%, if we treat off-target and polar effects independently, but the real false positive rate should be much lower than this number.

False negatives can arise from inefficient TF repression (i.e., not creating an effective perturbation). CRISPRi repression tested in minimal glucose condition (Supplementary Note 2) showed that 4 out of 52 TFs (41+14-3; 41 from promoters directly targeted by sgRNAs, 14 from RT-qPCR, and 3 shared by both methods) were not significantly repressed by CRISPRi, which gives an expected false negative rate about 8%, if we equally treat every TF.

Supplementary Note 6. Choice of replication origin

The replication origin of the reporter plasmid was carefully chosen. Preliminary tests performed using pMB1 (~20 copies per cell) as the replication origin showed that a few promoter-reporter strains, such as P_{glyA} and P_{metA}, cloned to this plasmid could not grow in the M9 medium. Because *glyA* and *metA* are essential genes when cells are growing in the M9 medium[15], we speculated that the high copy number of promoter sequences in the pMB1 plasmid sequestered too many regulators, thus leading to impaired cell growth. When low copy number plasmids pSC101 (~4 copies) and pBAC (single copy) were used, strains with P_{glyA} and P_{metA} reporter plasmids can grow in the M9 medium (Supplementary Fig. 15). To ensure high sensitivity in the fluorescence measurements, a plasmid with the pSC101 replication origin was used for library cloning.

Supplementary Figures:

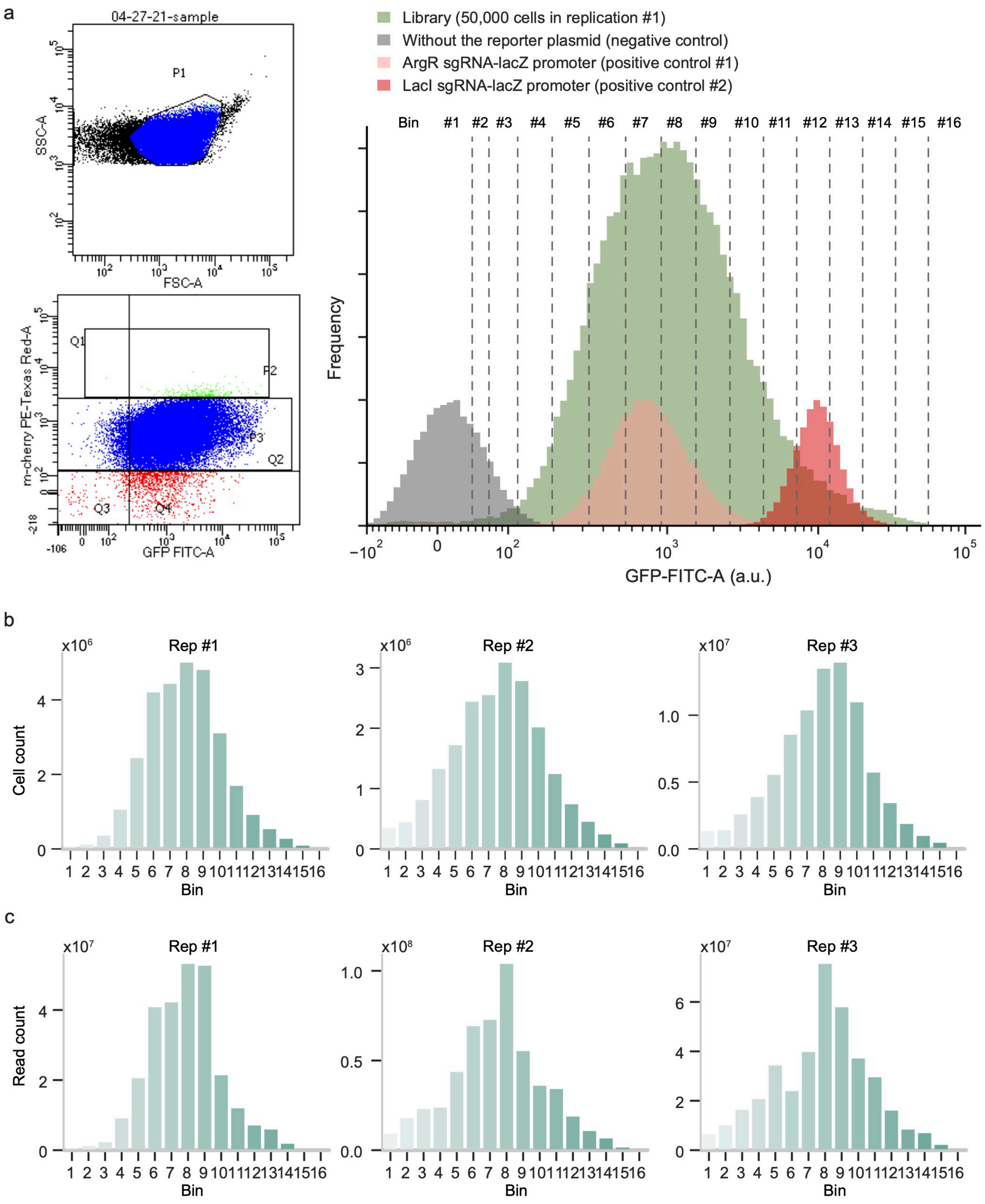

Supplementary Figure 1. FACS gate settings and sequencing results of PPTP-seq.

a FACS gate setting for the combinatorial library. The library was first selected for a homogenous cell population (P1) by side-angle scattering (SSC-A) and forward-angle scattering (FSC-A). Subpopulation (Q2) from P1 were selected based on GFP and mCherry fluorescence. Q2 population were finally sorted into 16 equally sized contiguous bins according to their GFP fluorescence intensity on a log scale. Two positive controls and a negative control indicated that strains with different GFP intensities were sorted into different bins. **b** The number of cells collected in each bin for three replicates. **c** The number of reads sequenced from each bin for three replicates. Source data are provided in Supplementary Data 3.

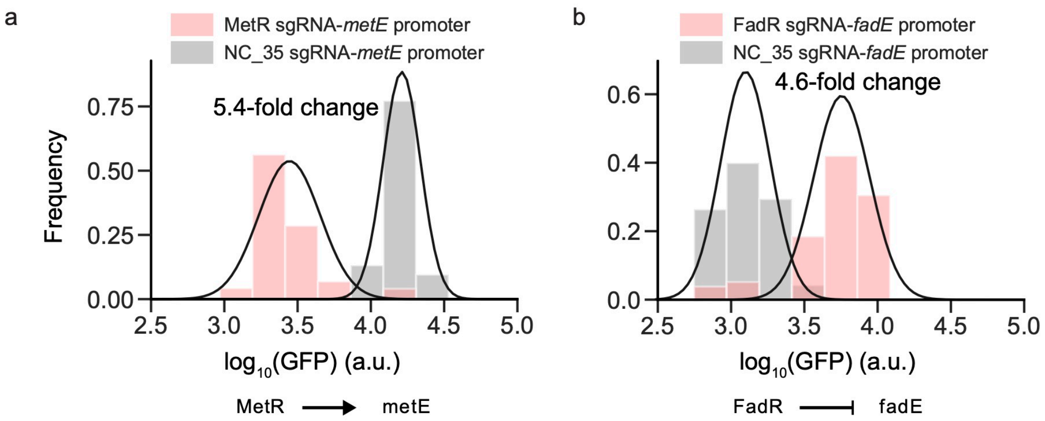

Supplementary Figure 2. Representative PPTP-seq results.

a and **b** Reconstructed GFP distributions expressed from *metE* promoter (**a**) and *fadE* promoter (**b**) with a non-targeting sgRNA (grey) or a sgRNA targeting to their corresponding regulators (red). The bar plots show the estimated cell fraction in each bin for a sgRNA-promoter pair. A log-normal distribution was fitted into the estimated cell fractions for each sgRNA-promoter pair using maximum likelihood estimation. MetR is a known activator of the *metE* promoter and FadR is a known repressor of the *fadE* promoter.

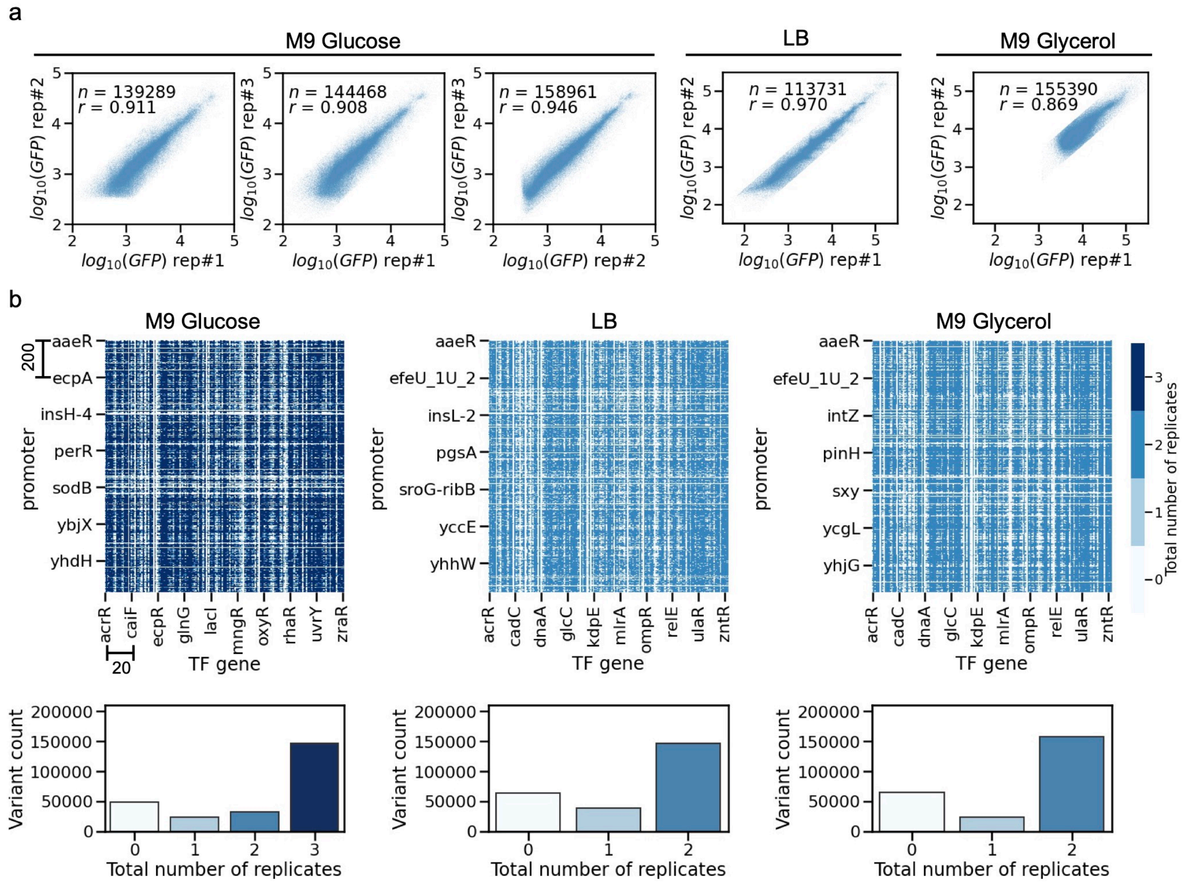

Supplementary Figure 3. Data quality of PPTP-seq performed at different conditions.

a Evaluation on the biological reproducibility of PPTP-seq performed using M9 glucose, LB, and M9 glycerol media. Measurements with a standard deviation of the log(GFP) larger than 0.7 were discarded. **b** Coverage of PPTP-seq performed using M9 glucose, LB, and M9 glycerol media. In the heatmap, each row represents a promoter, and each column represents a TF.

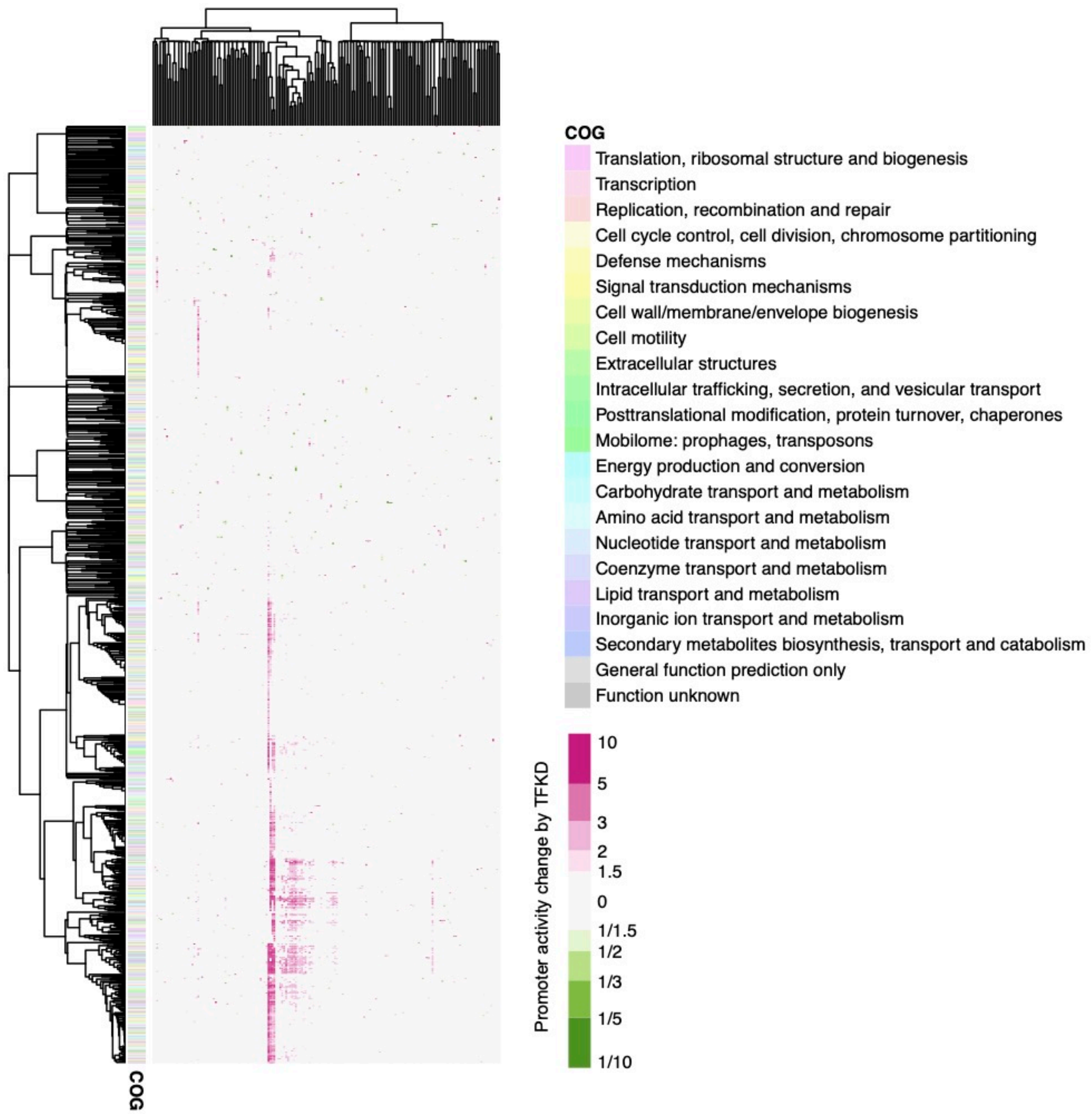

Supplementary Figure 4. Promoter activity fold changes by TFKD.

The matrix was hierarchically clustered in both dimensions. Hierarchical clustering was performed using the average linkage method with the distance matrix for Pearson correlation of promoter activity fold changes by TFKD. Promoters without activity changes under any TFKD condition were not shown. Missing data and non-significant fold changes were set zero in the matrix. Source

data are provided as a Source Data file (the same as Figure 1d). The COG annotation is from COG database 2020 update [https://ftp.ncbi.nih.gov/pub/COG/COG2020/data/].

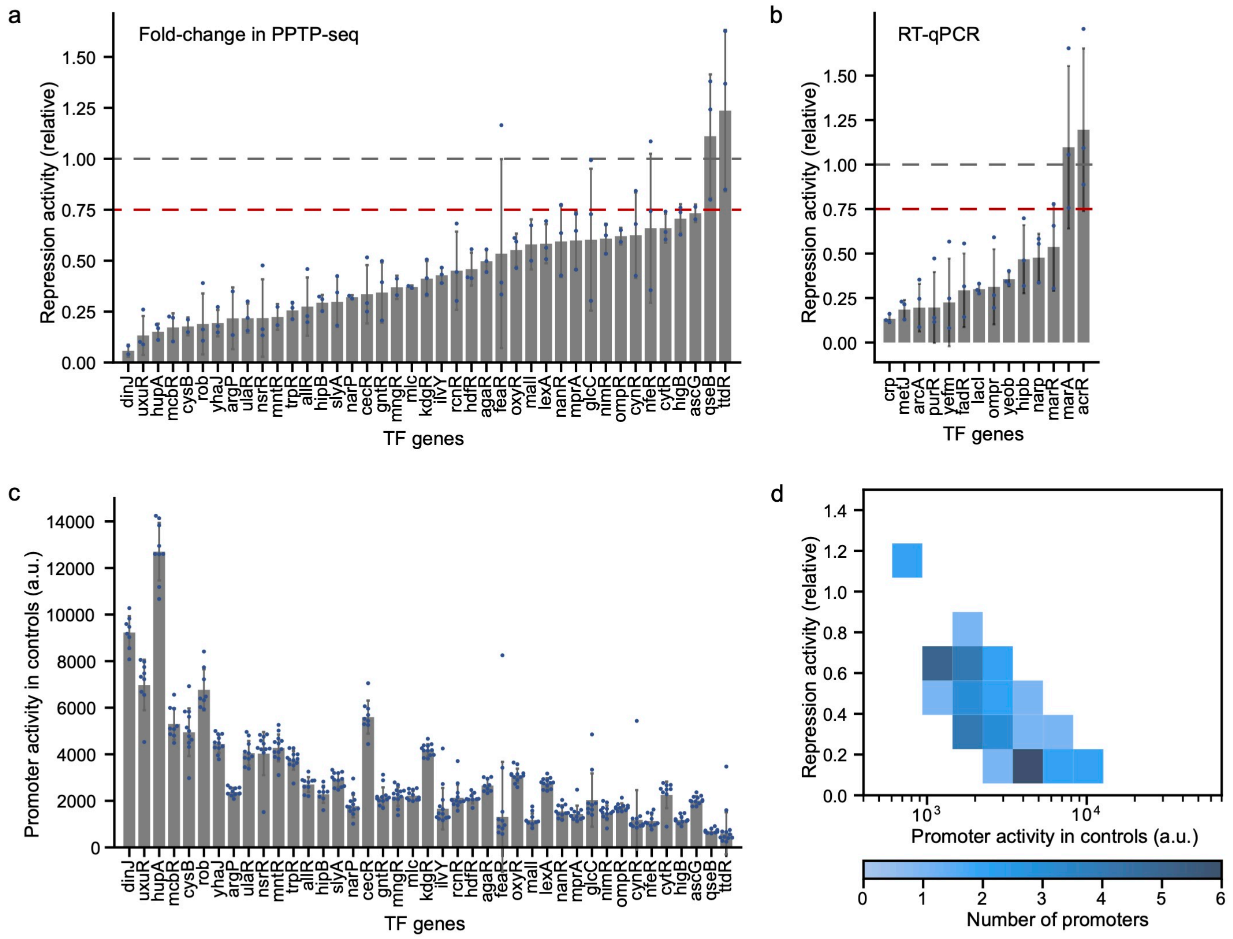

Supplementary Figure 5. Evaluation of CRISPRi repression efficiency.

a and **b** Repression of TF genes by CRISPRi measured by PPTP-seq (**a**) and RT-qPCR (**b**). Columns show relative activities from three biological replicates. Error bars indicate standard deviation. **c** Relative TF expression level as measured by their promoter activities in PPTP-seq using the strain with non-targeting control sgRNA. **d** Relationship between CRISPRi repression activity and TF promoter activity without CRISPRi repression. Source data are provided as a Source Data file.

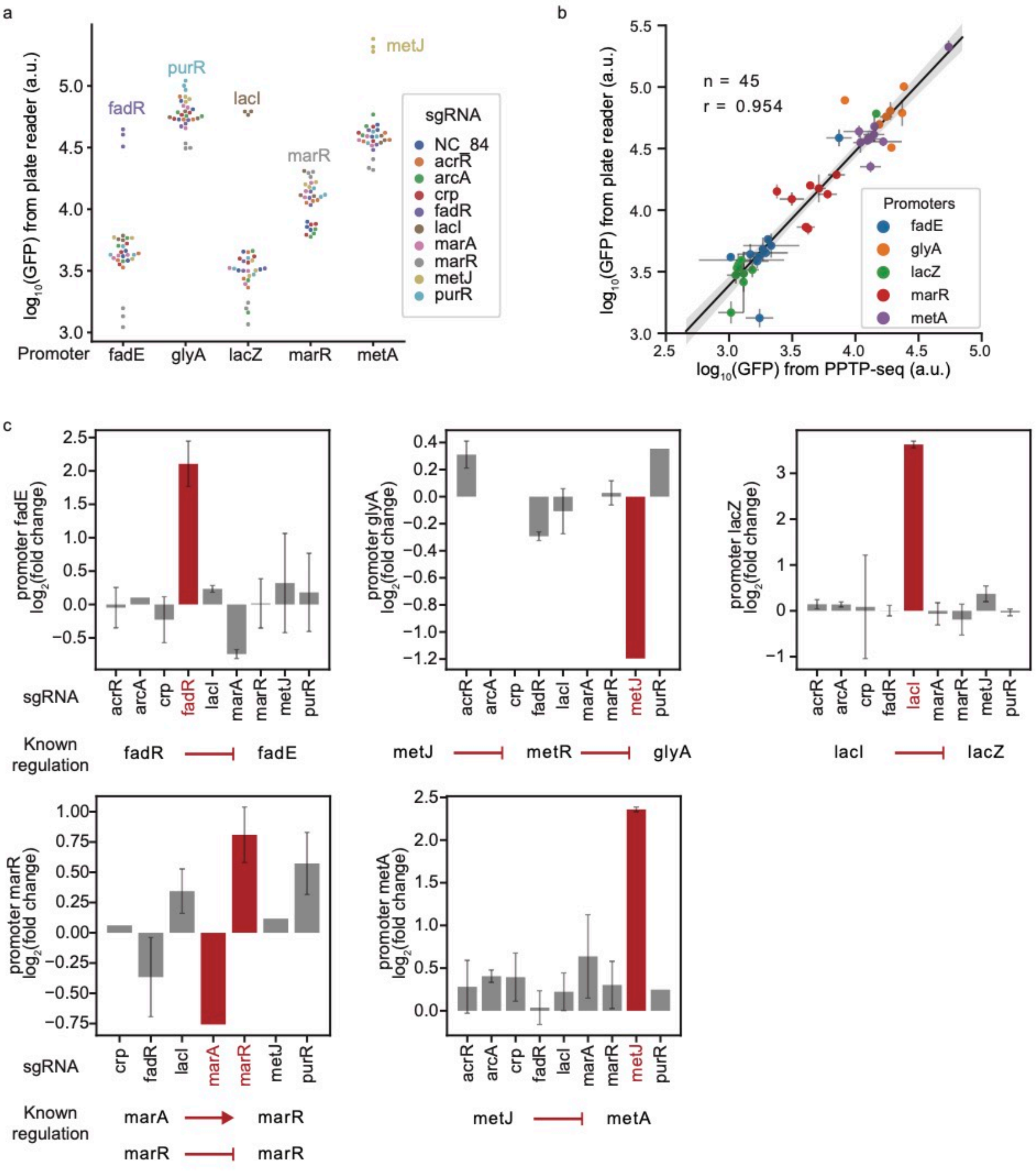

Supplementary Figure 6. Benchmarks of PPTP-seq on five random promoters.

a Individual promoter activity measured from whole cell fluorescence using a plate reader. Three replicates were performed for each strain on different days. **b** Correlation between individual measurements by the plate reader and pooled measurements by PPTP-seq. **c** Promoter activity changes relative to the negative control measured by PPTP-seq matched known regulations in the

RegulonDB. Data are presented as means ± SD of at least two replicates from different days. a.u., arbitrary units. Source data are provided as a Source Data file.

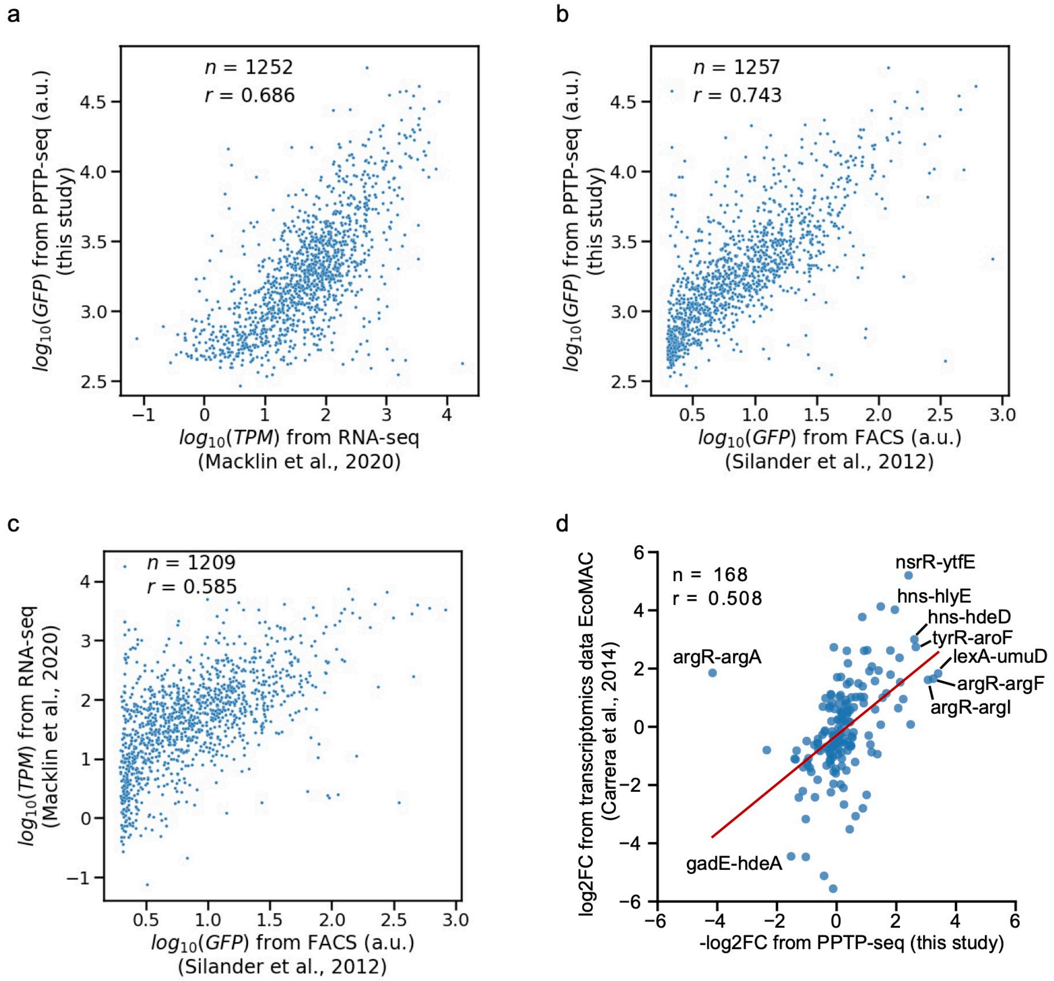

Supplementary Figure 7. Correlation of PPTP-seq data with published data sets.

a and **b** Promoter activity measured from PPTP-seq were compared to transcript level measured from RNA-seq dataset in Macklin et al.[16] (**a**) and promoter activity measured by flow cytometry in Silander et al.[17] (**b**). **c** As a reference, data in Macklin et al. were compared to data in Silander et al. Correlation in **c** is weaker than correlations shown in **a** and **b**. **d** Comparison of fold changes at the log2 scale (log2FC) measured from PPTP-seq to log2FC obtained from EcoMAC microarray[18]. Red line represents a fitting result of linear regression. For each plot, number of values (n) and Pearson's correlation coefficient (r) are displayed. TPM, transcript per million. Source data are provided as a Source Data file.

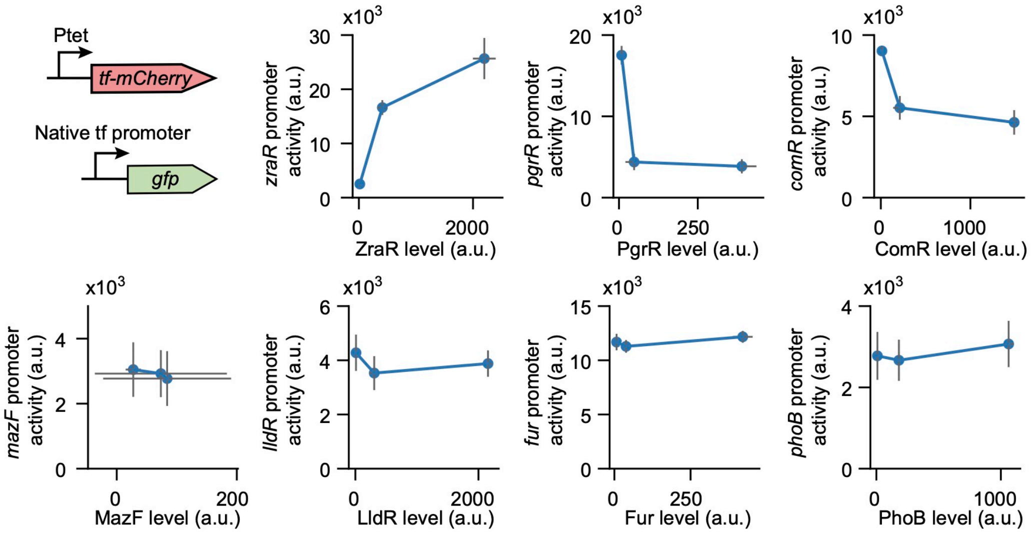

Supplementary Figure 8. Validation of TF auto-regulatory responses

Validation was performed in TF-tunable strains, where the endogenous TF gene copy is deleted. TF expression levels were measured using mCherry fluorescence, and their promoter activities were quantified by GFP fluorescence. Data are presented as means ± SD of three replicates from different days. a.u., arbitrary units. Source data are provided as a Source Data file.

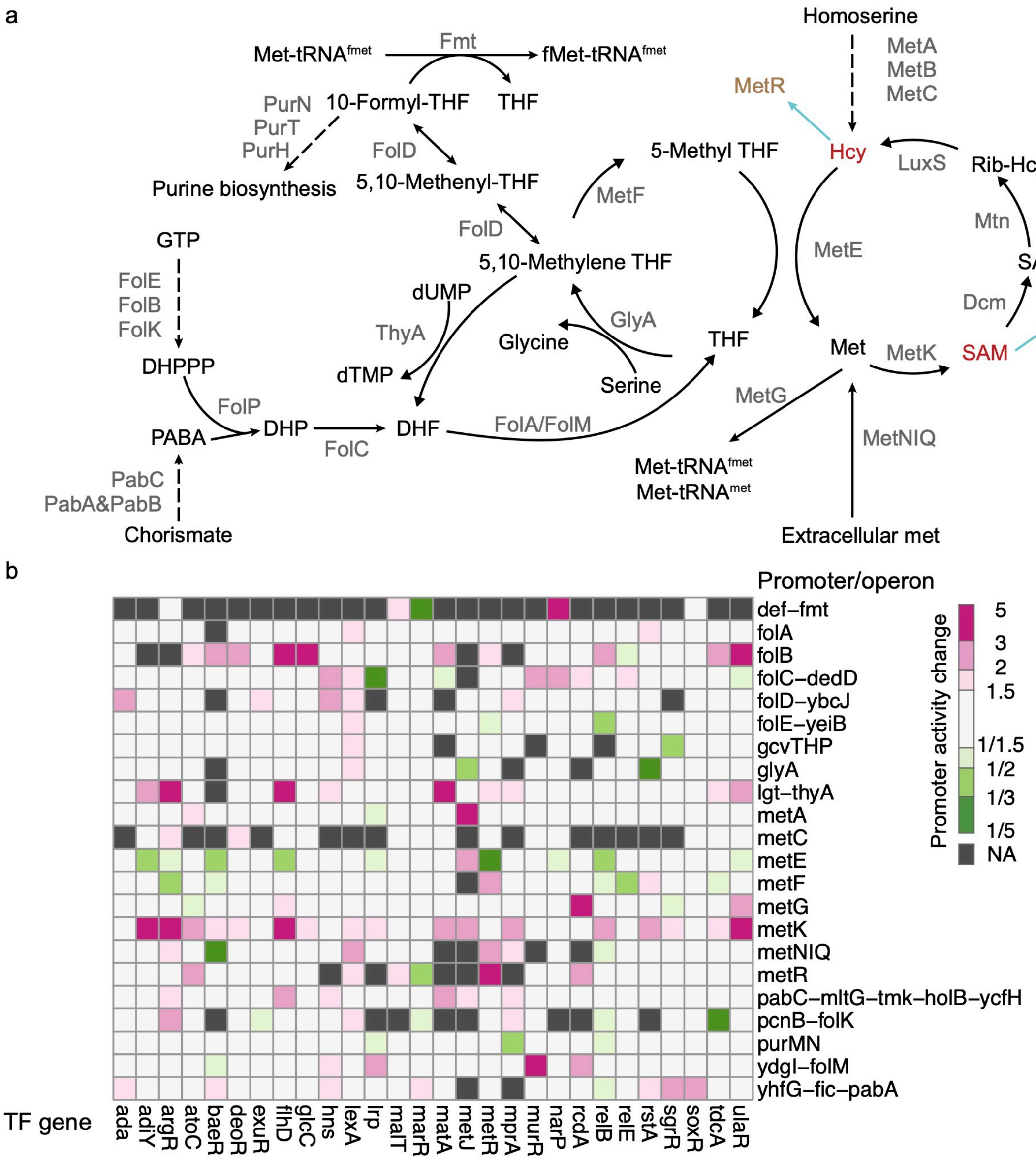

Supplementary Figure 9. Promoter activity changes in one-carbon metabolism.

a Pathway map of one-carbon metabolism. Hcy and SAM control the activity of MetR and MetJ, respectively. NA, not applicable; GTP, Guanosine-5'-triphosphate; DHPPP, 6-hydroxymethyl-7,8-dihydropterin pyrophosphate; PABA, *para*-aminobenzoic acid; DHP, dihydropteroate; DHF, dihydrofolate; THF, tetrahydrofolate; dUMP, deoxyuridine monophosphate; dTMP, deoxythymidine monophosphate; Met, L-methionine; fMet, N-formylmethionine; Hcy, L-

homocysteine; SAM, S-adenosylmethionine; SAH, S-adenosylhomocysteine; Rib-Hcy, S-ribosyl-L-homocysteine. **b** Heatmap of promoter activity changes.

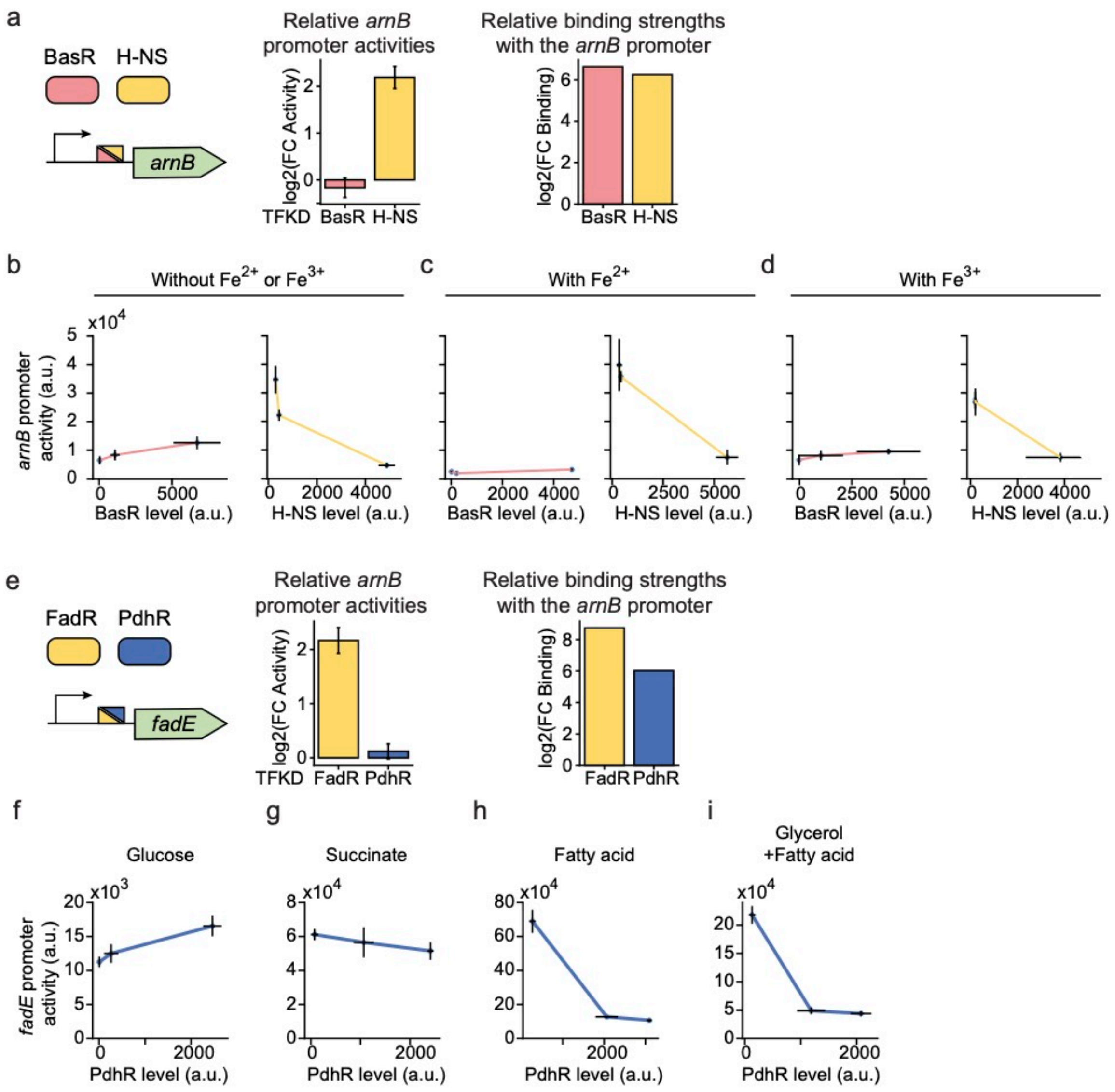

Supplementary Figure 10. Context-dependent regulatory functions for promoters controlled by multiple TFs.

a Regulatory logic for the *arnB* promoter. Log2(FC Activity) is the promoter activity change by TFKD in PPTP-seq results in minimal glucose medium. Log2(FC Binding) is binding strength in DAP-seq results. **b** *arnB* promoter activity at different BasR or H-NS concentrations in glucose minimal medium without Fe^{2+} or Fe^{3+}. **c** and **d** *arnB* promoter activity at different BasR or H-NS concentrations in glucose minimal medium in presence of Fe^{2+} (**c**) or Fe^{3+} (**d**). **e** Regulatory logic

for the *fadE* promoter. **f-i** *fadE* promoter activity at different PdhR concentrations in M9 medium with different carbon sources, including glucose (**f**), succinate (**g**), fatty acid (**h**), and glycerol plus fatty acid (**i**). Promoter activities at different TF expression levels were measured using TF-tunable strains. Data are presented as means ± SD of at least two replicates from different days. NA, not applicable; a.u., arbitrary units. Source data are provided as a Source Data file.

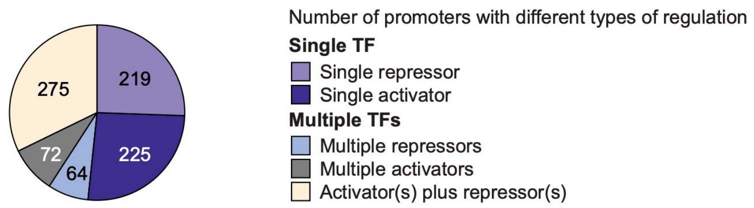

Supplementary Figure 11. Various types of regulatory architectures on promoters according to RegulonDB.

The number of promoters belonging to each type is indicated.

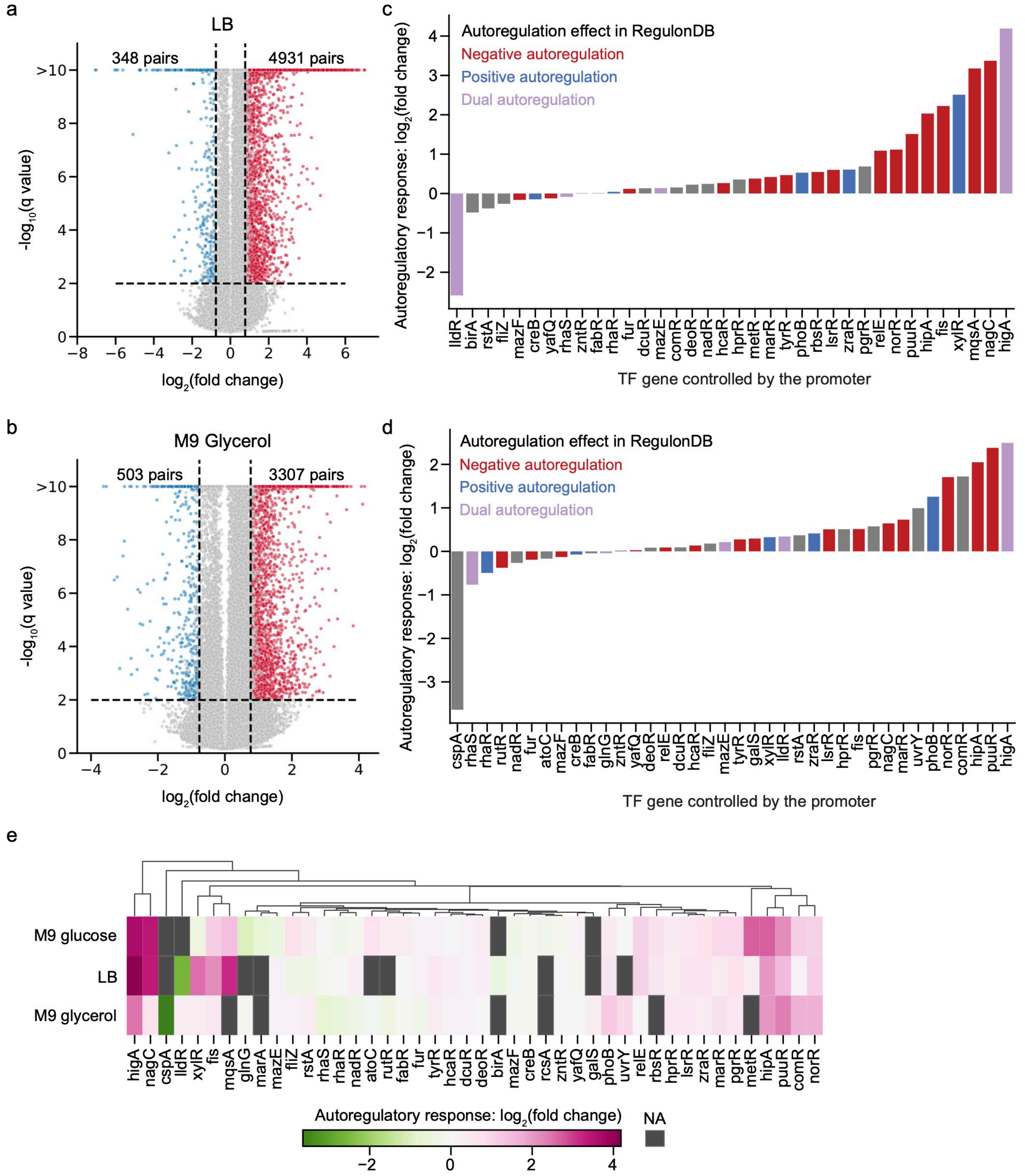

Supplementary Figure 12. Promoter responses by TFKD in LB and M9 glycerol media.

a and **b** Promoter activity changes by TFKD in LB (**a**) and M9 glycerol (**b**) media. Dashed lines indicate cutoffs for statistically significant ($q < 0.01$) and substantial (>1.7-fold change) effects.

Each dot represents a TF-promoter pair. Up-regulation and down-regulation by TFKD are shown in red and blue, respectively. **c** and **d** Autoregulation of TFs identified by PPTP-seq in LB (**c**) and M9 glycerol (**d**) media. Promoter activity fold changes upon the knockdown of TF controlled by the promoter. **e** Comparison of auto-regulatory responses measured by PPTP-seq in minimal glucose, LB, and minimal glycerol media.

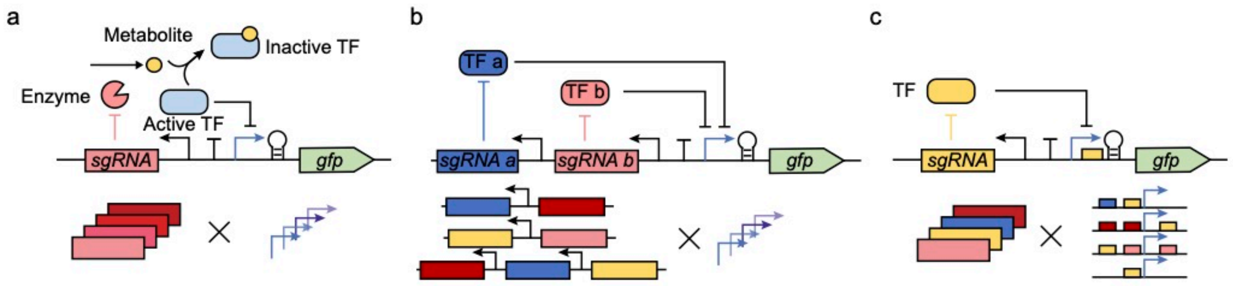

Supplementary Figure 13. Potential extensions of PPTP-seq

a, Changing sgRNA targets to enzymes and transporters to study metabolism-related regulation. **b**, Using sgRNA arrays to study effects from knockdown of multiple TFs. **c**, Replacing the native promoter library with a synthetic promoter library containing various binding site combinations to study detailed regulatory mechanism of complex promoters.

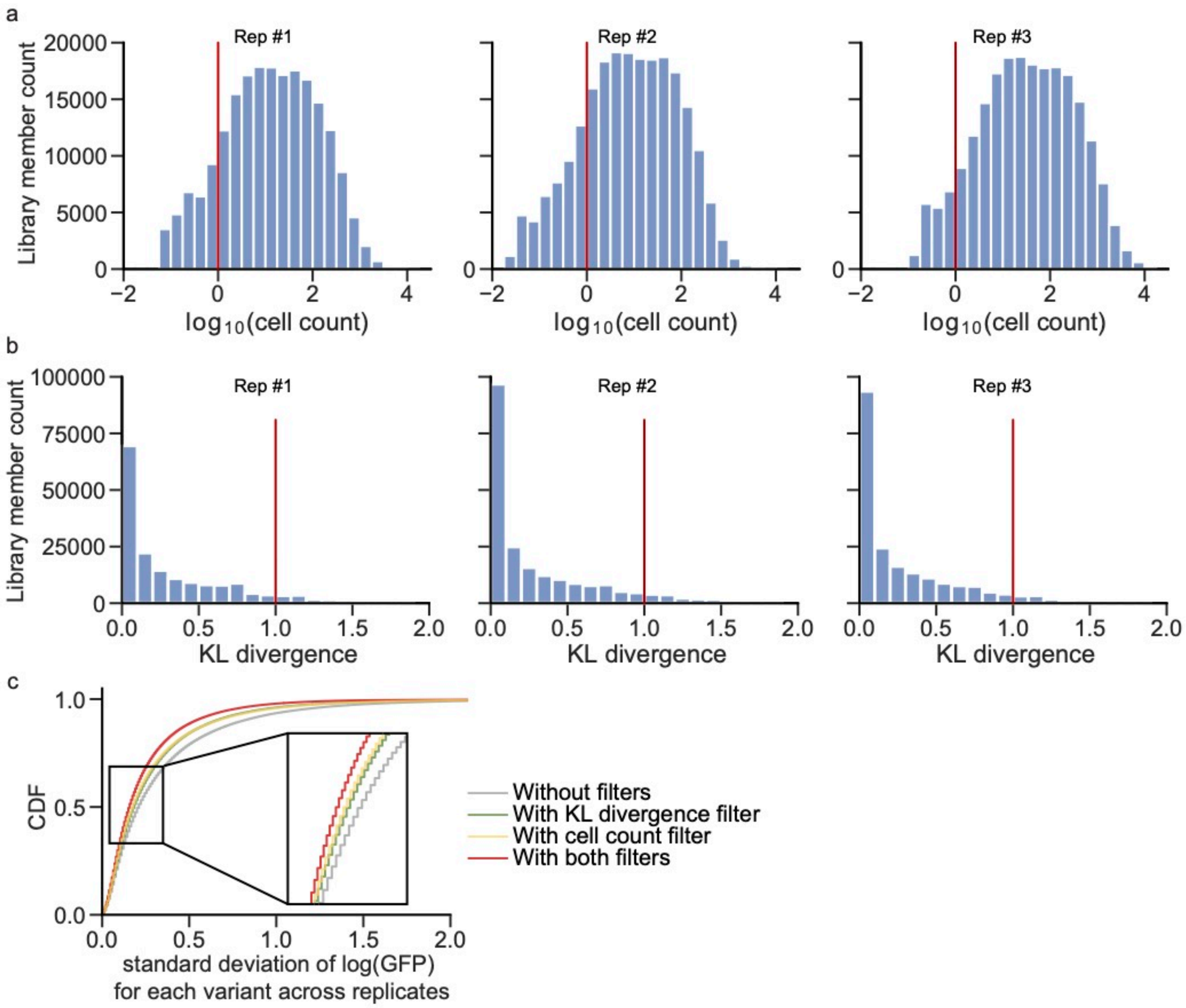

Supplementary Figure 14. Data quality filters improved data consistency

a, Estimated cell count distributions of the library. **b**, KL divergence distributions of the fitting result for each member in the library. **c**, Cumulative density function (CDF) of the standard deviation of log(GFP) for data treated by different filtering rules. Applying the KL divergence filter and cell count filter reduced the standard deviation.

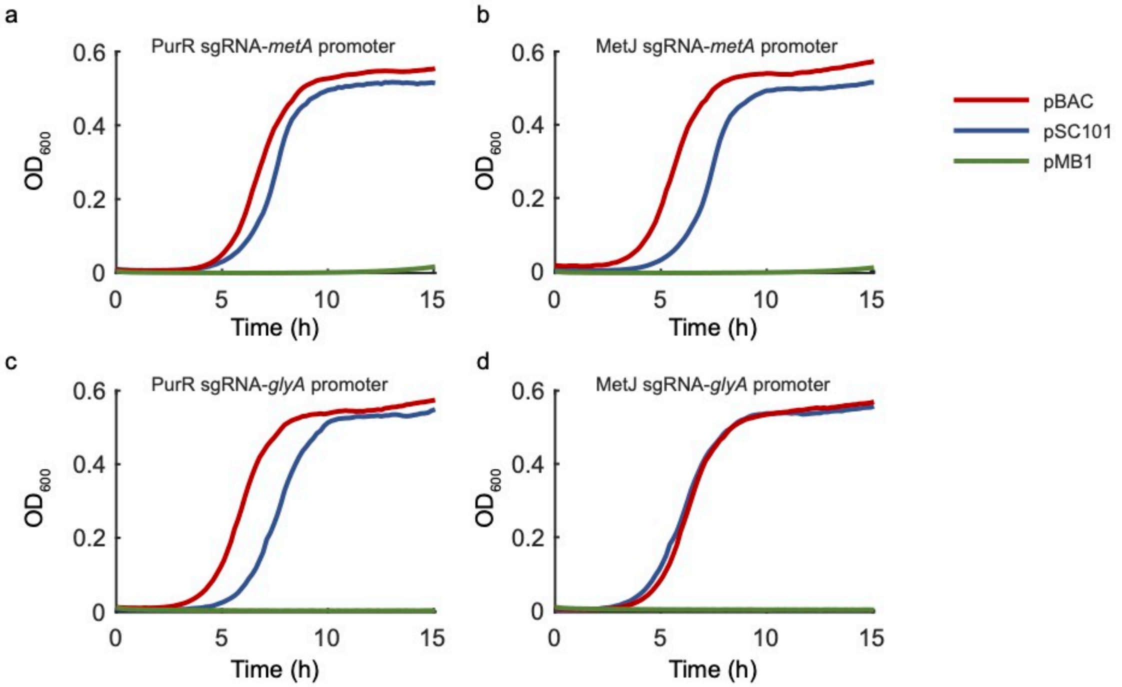

Supplementary Figure 15. Effects of plasmid copy number on cell growth

a-d, Growth curves of reporter plasmids of four TF-promoter pairs, including PurR-*metA* (**a**), MetJ-*metA* (**b**), PurR-*glyA* (**c**), and MetJ-*glyA* (**d**) constructed with different replication origins. Strains containing a plasmid with pMB1 origin cannot grow in the M9 glucose medium during the first 15 hours.

Supplementary Tables:

Supplementary Table 1. Regulatory responses identified from PPTP-seq and known from RegulonDB

	M9 Glucose		LB		M9 Glycerol	
Binding evidence	With	Without	With	Without	With	Without
In RegulonDB	78	0	53	1	43	0
New responses	403	3577	397	4828	230	3537
Total	481	3577	450	4829	373	3537
PPTP-seq significant responses	4058		5279		3810	

Supplementary Table 2. The number of responses for selected TFs in different conditions

TF	M9 Glucose	LB	M9 glycerol	known effector
birA	62	162	26	biotinyl-5'-adenylate
glcC	149	202	91	glycolate
hypT	107	246	66	hypochlorous acid
melR	70	192	22	melibiose
narL	65	201	16	nitrate (NarX)
rcnR	44	162	13	Ni(II) and Co(II)
ulaR	345	442	271	L-ascorbate-6-phosphate
Sum	842	1607	505	

Supplementary References

1. Rintoul, M. R. *et al.* Regulation of the Escherichia coli Allantoin Regulon: Coordinated Function of the Repressor AllR and the Activator AllS. *J. Mol. Biol.* **324**, 599–610 (2002).

2. Pellicer, M. T. *et al.* Cross-induction of glc and ace Operons ofEscherichia coli Attributable to Pathway Intersection. *J. Biol. Chem.* **274**, 1745–1752 (1999).

3. Elowitz, M. B. & Leibier, S. A synthetic oscillatory network of transcriptional regulators. *Nature* **403**, 335–338 (2000).

4. Winfield, M. D. & Groisman, E. A. Phenotypic differences between Salmonella and Escherichia coli resulting from the disparate regulation of homologous genes. *Proc. Natl. Acad. Sci. U.S.A.* **101**, 17162–17167 (2004).

5. Quail, M. A. & Guest, J. R. Purification, characterization and mode of action of PdhR, the transcriptional repressor of the pdhR-aceEF-lpd operon of Escherichia coli. *Mol. Microbiol.* **15**, 519–29 (1995).

6. Xu, Y. F., Amador-Noguez, D., Reaves, M. L., Feng, X. J. & Rabinowitz, J. D. Ultrasensitive regulation of anapleurosis via allosteric activation of PEP carboxylase. *Nat. Chem. Biol.* **8**, 562–568 (2012).

7. Cronan, J. E. In vivo evidence that acyl coenzyme A regulates DNA binding by the Escherichia coli FadR global transcription factor. *J. Bacteriol.* **179**, 1819–1823 (1997).

8. Zhang, F., Carothers, J. M. & Keasling, J. D. Design of a dynamic sensor-regulator system for production of chemicals and fuels derived from fatty acids. *Nat. Biotechnol.* **30**, 354–359 (2012).

9. Xiao, Y., Bowen, C. H., Liu, D. & Zhang, F. Exploiting nongenetic cell-to-cell variation for enhanced biosynthesis. *Nat. Chem. Biol.* **12**, 339–344 (2016).

10. Hartline, C. J., Mannan, A. A., Liu, D., Zhang, F. & Oyarzún, D. A. Metabolite sequestration enables rapid recovery from fatty acid depletion in Escherichia coli. *MBio* **11**, 1–11 (2020).

11. Cui, L. *et al.* A CRISPRi screen in E. coli reveals sequence-specific toxicity of dCas9. *Nat. Commun.* **9**, 1–10 (2018).

12. Wang, T. *et al.* Pooled CRISPR interference screening enables genome-scale functional genomics study in bacteria with superior performance-net. *Nat. Commun.* **9**, (2018).

13. Peters, J. M. *et al.* A comprehensive, CRISPR-based functional analysis of essential genes in bacteria. *Cell* **165**, 1493–1506 (2016).

14. Peters, J. M. *et al.* Bacterial CRISPR: Accomplishments and prospects. *Curr. Opin. Microbiol.* **27**, 121–126 (2015).

15. Baba, T. *et al.* Construction of Escherichia coli K-12 in-frame, single-gene knockout mutants: The Keio collection. *Mol. Syst. Biol.* **2**, 2006.0008 (2006).

16. Macklin, D. N. *et al.* Simultaneous cross-evaluation of heterogeneous E. coli datasets via mechanistic simulation. *Science* **369**, eaav3751 (2020).

17. Silander, O. K. *et al.* A genome-wide analysis of promoter-mediated phenotypic noise in Escherichia coli. *PLoS Genet.* **8**, 836–845 (2012).

18. Carrera, J. *et al.* An integrative, multi-scale, genome-wide model reveals the phenotypic landscape of E scherichia coli. *Mol. Syst. Biol.* **10**, 735 (2014).
