# Supplementary material for: Genome-wide promoter responses to CRISPR perturbations of regulators reveal regulatory networks in Escherichia coli
Source: Nat Commun. 2023 Sep 16;14:5757. doi: 10.1038/s41467-023-41572-4 (PMC10505187; doi:10.1038/s41467-023-41572-4)
Supplement: Supplementary file 2 — Reporting Summary [file 41467_2023_41572_MOESM2_ESM.pdf]

nature portfolio

Corresponding author(s): Fuzhong Zhang
Last updated by author(s): Sep 1, 2023

Reporting Summary

Nature Portfolio wishes to improve the reproducibility of the work that we publish. This form provides structure for consistency and transparency in reporting. For further information on Nature Portfolio policies, see our Editorial Policies and the Editorial Policy Checklist.

Statistics

For all statistical analyses, confirm that the following items are present in the figure legend, table legend, main text, or Methods section.

n/a	Confirmed	
☐	☒	The exact sample size (*n*) for each experimental group/condition, given as a discrete number and unit of measurement
☐	☒	A statement on whether measurements were taken from distinct samples or whether the same sample was measured repeatedly
☐	☒	The statistical test(s) used AND whether they are one- or two-sided<br>*Only common tests should be described solely by name; describe more complex techniques in the Methods section.*
☒	☐	A description of all covariates tested
☐	☒	A description of any assumptions or corrections, such as tests of normality and adjustment for multiple comparisons
☐	☒	A full description of the statistical parameters including central tendency (e.g. means) or other basic estimates (e.g. regression coefficient) AND variation (e.g. standard deviation) or associated estimates of uncertainty (e.g. confidence intervals)
☐	☒	For null hypothesis testing, the test statistic (e.g. *F*, *t*, *r*) with confidence intervals, effect sizes, degrees of freedom and *P* value noted<br>*Give P values as exact values whenever suitable.*
☒	☐	For Bayesian analysis, information on the choice of priors and Markov chain Monte Carlo settings
☒	☐	For hierarchical and complex designs, identification of the appropriate level for tests and full reporting of outcomes
☐	☒	Estimates of effect sizes (e.g. Cohen's *d*, Pearson's *r*), indicating how they were calculated

Our web collection on statistics for biologists contains articles on many of the points above.

Software and code

Policy information about availability of computer code

Data collection	FACSDiva 8.0.1 for cell sorting; Bowtie2 v2.3.5 and BEDTools v2.29.2 for read mapping; a custom Python script for mapped read count processing and fitting. All scripts are available via Github (doi.org/10.5281/zenodo.8309683).
Data analysis	Scripts for NGS data processing and Jupyter Notebooks for data analysis are available at doi.org/10.5281/zenodo.8309683.

For manuscripts utilizing custom algorithms or software that are central to the research but not yet described in published literature, software must be made available to editors and reviewers. We strongly encourage code deposition in a community repository (e.g. GitHub). See the Nature Portfolio guidelines for submitting code & software for further information.

Data

Policy information about availability of data

All manuscripts must include a data availability statement. This statement should provide the following information, where applicable:

- Accession codes, unique identifiers, or web links for publicly available datasets
- A description of any restrictions on data availability
- For clinical datasets or third party data, please ensure that the statement adheres to our policy

The PPTP-seq generated in this study have been deposited in the GEO database under accession code GSE213624 [https://www.ncbi.nlm.nih.gov/geo/query/acc.cgi?acc=GSE213624]. The plate reader and RT-qPCR data generated in this study are provided in the Source Data file. The RNA-seq and microarray data used in this study are available at GitHub [https://github.com/CovertLab/wcEcoli/tree/master/reconstruction/ecoli/flat]. The TF binding datasets used in this study are available at RegulonDB high throughput collection [https://regulondb.ccg.unam.mx/menu/integrated_views_and_tools/regulondb-ht_datasets/index.jsp].

Research involving human participants, their data, or biological material

Policy information about studies with human participants or human data. See also policy information about sex, gender (identity/presentation), and sexual orientation and race, ethnicity and racism.

Reporting on sex and gender	N/A
Reporting on race, ethnicity, or other socially relevant groupings	N/A
Population characteristics	N/A
Recruitment	N/A
Ethics oversight	N/A

Note that full information on the approval of the study protocol must also be provided in the manuscript.

Field-specific reporting

Please select the one below that is the best fit for your research. If you are not sure, read the appropriate sections before making your selection.

[x] Life sciences [ ] Behavioural & social sciences [ ] Ecological, evolutionary & environmental sciences

For a reference copy of the document with all sections, see nature.com/documents/nr-reporting-summary-flat.pdf

Life sciences study design

All studies must disclose on these points even when the disclosure is negative.

Sample size	No sample size calculation was performed. The sample size in this study is determined by comparing published studies using similar technology (McClune et al., Nature 2019; de Boer et al., Nat Biotechnol 2020; Kotopka et al., Nat Commun 2020) . Two or three replicates were used in these studies and results were consistent.
Data exclusions	We fitted the sort-seq data of each variant to log normal distribution and calculated the Kullback-Leibler (KL) divergence between inferred distribution from maximum likelihood estimation and the measured distribution. To control the fitting quality, fitted parameters of variants with any of the following features were excluded from subsequent analysis: i) the variants with mean GFP intensity not within our detection limits (between 10^1.5 and 10^5 for the M9 glucose growth condition and 10^1.5 to 10^5.5 for the LB and M9 glycerol growth conditions); ii) variants with estimated cell counts less than 1; iii) variants with the KL divergence greater than 1; iv) variants with all cells in a single gate. All sgRNA-promoter pairs that contain a sgRNA sequence directly targets the promoter region on the plasmid were excluded from differential expression analysis. For each promoter, outliers in negative control samples were excluded using the interquartile range (IQR) method.
Replication	We performed sort-seq experiments in three biological replicates for M9 glucose condition and two biological replicates for M9 glycerol and LB conditions to assess the reproducibility of these measurements. All attempts at replication were successful. The means and standard deviations between replicates were calculated and used in statistical analysis.
Randomization	This study does not involve subjects that require randomization.
Blinding	This study does not involve procedures that require blinding.

Reporting for specific materials, systems and methods

We require information from authors about some types of materials, experimental systems and methods used in many studies. Here, indicate whether each material, system or method listed is relevant to your study. If you are not sure if a list item applies to your research, read the appropriate section before selecting a response.

Materials & experimental systems

n/a	Involved in the study
☒	☐ Antibodies
☒	☐ Eukaryotic cell lines
☒	☐ Palaeontology and archaeology
☒	☐ Animals and other organisms
☒	☐ Clinical data
☒	☐ Dual use research of concern
☒	☐ Plants

Methods

n/a	Involved in the study
☒	☐ ChIP-seq
☐	☒ Flow cytometry
☒	☐ MRI-based neuroimaging

Flow Cytometry

Plots

Confirm that:

☒ The axis labels state the marker and fluorochrome used (e.g. CD4-FITC).

☒ The axis scales are clearly visible. Include numbers along axes only for bottom left plot of group (a 'group' is an analysis of identical markers).

☒ All plots are contour plots with outliers or pseudocolor plots.

☒ A numerical value for number of cells or percentage (with statistics) is provided.

Methodology

Sample preparation	E. coli cell cultures were grown in a target medium (either M9 glucose, M9 glycerol, or LB medium) and induced with 1µm aTc. The induced cells were grown at 37°C until OD600 reached 0.1-0.2. At this point, the cell cultures were supplemented with 250 µg/mL chloramphenicol to halt protein production and were kept on ice until sorting.
Instrument	FACSAria II (for cells grown in the M9 glucose) or FACSMelody (for cells grown in LB and M9 glycerol media) cell sorters (BD Biosciences)
Software	BD FACSDiva (for data from FACSAria II) and BD FACSChorus (for data from FACSMelody)
Cell population abundance	29.0 million, 20.3 million, and 84.5 million sorted cells were collected in M9 glucose condition in three replicates respectively.40.3 million and 41.5 million sorted cells were collected in LB condition in two replicates respectively. 32.8 million and 32.2 million sorted cells were collected in M9 glycerol condition in two replicates respectively. The number of cells was determined by the number of events recorded in the gate.
Gating strategy	To control extrinsic protein production noise, events were gated around the mean fluorescence of mCherry, which is constitutively expressed on the reporter plasmid. Cells were sorted into 16 equally sized contiguous bins according to their GFP fluorescence intensity (FITC-A) on a log scale.

☒ Tick this box to confirm that a figure exemplifying the gating strategy is provided in the Supplementary Information.
