# Supplementary material for: Genome-wide promoter responses to CRISPR perturbations of regulators reveal regulatory networks in Escherichia coli
Source: Nat Commun. 2023 Sep 16;14:5757. doi: 10.1038/s41467-023-41572-4 (PMC10505187; doi:10.1038/s41467-023-41572-4)
Supplement: Supplementary file 14 — Description of Additional Supplementary Files [file 41467_2023_41572_MOESM14_ESM.pdf]

Description of Additional Supplementary Data Files

File Name: Supplementary Data 1.
Description: TF library.

File Name: Supplementary Data 2.
Description: Promoters analyzed in the study.

File Name: Supplementary Data 3.
Description: Statistics in PPTP-seq.

File Name: Supplementary Data 4.
Description: KEGG and GO enrichment analysis. DAVID server was used to perform KEGG and GO enrichment analysis. Raw p values are calculated by one-sided Fisher's Exact test. Benjamini and Hochberg (2000) method is used to adjust p values.

File Name: Supplementary Data 5.
Description: TF perturbation-response network.

File Name: Supplementary Data 6.
Description: Response with binding evidence.

File Name: Supplementary Data 7.
Description: Regulatory responses for known regulatory interactions.

File Name: Supplementary Data 8.
Description: Primers. Index sequences used in NGS are highlighted in red.

File Name: Supplementary Data 9.
Description: sgRNA guide sequences.

File Name: Supplementary Data 10.
Description: Plasmids.

File Name: Supplementary Data 11.
Description: *E. coli* strains.
